# Collaborative hierarchy maintains cooperation in asymmetric games

**DOI:** 10.1038/s41598-018-23681-z

**Published:** 2018-03-29

**Authors:** Alberto Antonioni, María Pereda, Katherine A. Cronin, Marco Tomassini, Angel Sánchez

**Affiliations:** 1 Department of Economics, University College London, London, UK; 2 Grupo Interdisciplinar de Sistemas Complejos (GISC), Departamento de Matemáticas, Universidad Carlos III de Madrid, 28911 Leganés, Madrid Spain; 3 Institute for Biocomputation and Physics of Complex Systems (BIFI), University of Zaragoza, 50018 Zaragoza, Spain; 4 Unidad Mixta de Comportamiento y Complejidad Social, UC3M-UV-UZ (UMICCS), Leganés, Spain; 5 RWTH Aachen University, Chair for Computational Social Sciences and Humanities, Aachen, Germany; 6 Lester E. Fisher Center for the Study and Conservation of Apes, Lincoln Park Zoo, Chicago, IL USA; 7 Information Systems Department, Faculty of Business and Economics, University of Lausanne, CH-1015 Lausanne, Switzerland; 8 UC3M-BS Institute for Financial Big Data (IFiBiD), Universidad Carlos III de Madrid, 28903 Getafe, Madrid Spain

## Abstract

The interplay of social structure and cooperative behavior is under much scrutiny lately as behavior in social contexts becomes increasingly relevant for everyday life. Earlier experimental work showed that the existence of a social hierarchy, earned through competition, was detrimental for the evolution of cooperative behaviors. Here, we study the case in which individuals are ranked in a hierarchical structure based on their performance in a collective effort by having them play a Public Goods Game. In the first treatment, participants are ranked according to group earnings while, in the second treatment, their rankings are based on individual earnings. Subsequently, participants play asymmetric Prisoner’s Dilemma games where higher-ranked players gain more than lower ones. Our experiments show that there are no detrimental effects of the hierarchy formed based on group performance, yet when ranking is assigned individually we observe a decrease in cooperation. Our results show that different levels of cooperation arise from the fact that subjects are interpreting rankings as a reputation which carries information about which subjects were cooperators in the previous phase. Our results demonstrate that noting the manner in which a hierarchy is established is essential for understanding its effects on cooperation.

## Introduction

While cooperation is common in many species^1–3^, humans show this trait to a dramatically larger extent. This is evident in our unparalleled capability to cooperate with strangers in one-shot interactions and on a very large scale^4–6^. The emerging phenomenon of cooperation can involve working together with others in a mutually beneficial activity (i.e., a form of mutualism^7^), or incurring a costly action that helps others, thus reducing one’s own chances for survival under natural selection (i.e., altruism^8^). Both types of cooperation are ubiquitous in our daily lives, and constitute the pillar on which our society is built and functions^9^. However, for all its importance, the interplay between social structure and cooperative behavior in humans has received little attention^10^. In this context, it has been shown that active partner choice, i.e., the possibility to choose interaction partners at will or through assortment^11^, does lead to the establishment of cooperation^12,13^. However, past experimental work has rarely allowed social interaction, employing paradigms where all individuals were equal and anonymous, and choices motivated only by informational cues (i.e., reputation^14–16^,).

In most social interactions, some degree of asymmetry or inequality between positions in the network is a key factor. Particularly among primates, hierarchy or ranking is a determinant factor in the decision to work with another individual^1,10^. Even the mere presence of another, differently ranked subject has been shown to dramatically affect individuals’ performance^17^. Once not all individuals have the same strategic options and/or the consequences of their actions differ, those in a superior position can reap more benefits from cooperative actions at the expense of their partners, which in turn may lead the latter to stop cooperating. It has been shown recently^18^ that this is also the case in experiments with humans designed similarly to setups employed with primates^19,20^: Lower ranked subjects contribute less to a common goal when they benefit less than their partners. Interestingly, this appears to be due to the fact that when higher ranked subjects can coerce their counterparts into cooperating, they very often do so^21^ by resorting to so-called zero-determinant strategies^22,23^. It thus seems that the existence of a social structure, in the form of a ranking or a hierarchy, can have detrimental effects in the stability of cooperation among humans.

In this study we want to probe further into the interplay of social structure and cooperation by considering a different type of hierarchy. This is by no means an academic question in so far as hierarchies differ among primate species in their steepness and their linearity^24^, and organizations in our society come in very different flavors and structures^25^. Therefore, here we set out to study how cooperation is affected when hierarchies are not linear but instead there is more than one individual at each ranking level. Additionally, we contribute to the knowledge of cooperation on hierarchical structure by considering the case in which one’s ranking arises through competition with all others as in^18^, or through some amount of cooperation. We study these issues by means of a novel experimental design, which, as we will see below, allow us to shed light on hitherto unexplored facets of cooperative behavior. As shown in previous experimental works^8,26–29^, when pairwise interactions are repeated for a reasonably large number of rounds the mutual cooperation outcome is easier to achieve. Here, we employ a setting for testing the impact of hierarchy formation in short, but not one-shot, interactions, avoiding the direct reciprocity mechanism present for longer time encounters.

## Experimental Setup

The experimental setup we introduce in this work consists of three treatments, namely Selfish (or Competitive) Hierarchy (SH), Collaborative Hierarchy (CH) and Random Hierarchy (RH). The SH and CH treatments include two phases, named Phase I and Phase II, while RH treatments include only Phase II. All treatments involved exactly 24 participants per experimental session and participants’ scores were expressed in Experimental Currency Units (ECUs). However, only ECUs accumulated during Phase II were converted to real money at the end of the experimental session at an exchange rate of 80 ECUs = 1EUR. During Phase I participants in the SH and CH treatments acquired one hierarchy profile of four possible levels, called *A, B, C*, and *D*. Participants playing the RH treatment began the experiment directly at Phase II, with one of the four hierarchy profiles assigned to them at random. The four hierarchy profiles were equally represented in all the treatments, that is, there were six participants in each hierarchy profile. A translation of the exact experimental instructions can be found in the Supplementary Information (SI), see Section 1.

### Phase I: Hierarchy formation

During Phase I participants were randomly assigned to six groups of four participants and they played a Public Goods Game (PGG^30^,) within their groups for 15 rounds. The exact number of PGG rounds was unknown to participants who only knew that they had to play for at least 10 rounds. At the beginning of each round all participants decided how many points between 0 and 10 (one choice among options: 0, 2, 4, 6, 8 and 10) they wanted to contribute to the group common pool from their round endowment of 10 ECUs. The group common pool was then multiplied by two and then equally distributed to the four members of the group. Before proceeding to the following round participants received feedback on their group’s contribution level and on their individual payoff while they had no information on the situation of the other groups. In SH and CH treatments, hierarchy profiles were assigned at the end of Phase I according to the payoffs accumulated during the PGG. Phase I was skipped in RH treatments and hierarchy profiles were randomly distributed among all 24 participants. This last treatment was included because in previous experiments^18^ the effect of hierarchy did not depend on whether one’s own position was earned in a competition or received randomly, and we sought to measure whether this counter-intuitive phenomenon would replicate here.

**Figure 1:**
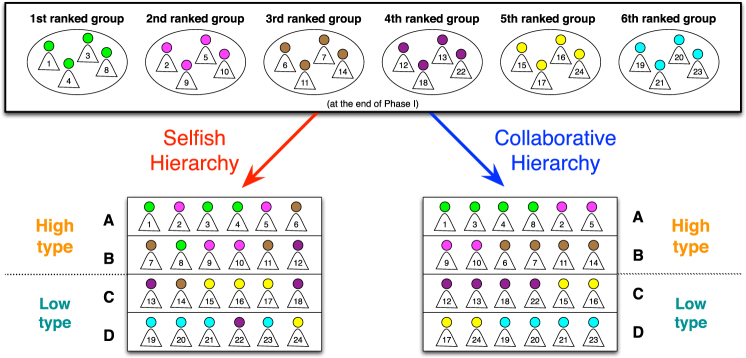
Sketch of the hierarchy assignment procedures. The 24 participants are ranked with respect to their cumulated payoff at the end of Phase I (numbers from 1 to 24) while the six groups are ranked according to the sum of individual payoffs of their group components. The four hierarchy profiles are divided into two class types: high (**A**,**B**) and low (**C**,**D**).

The difference between the SH and CH treatments arises in how the PGG is used to assign a position in the hierarchy. Both hierarchy assignment procedures are summarized in Fig. 1. In SH treatments the ranking of participants was computed according to the points each individual accumulated during Phase I. The six participants ranking highest were assigned to the first hierarchy profile (*A*), the next six participants to the second hierarchy profile (*B*), the next six participants to the third hierarchy profile (*C*), while the last six participants were assigned to the fourth hierarchy profile (*D*). On the other hand, in CH treatments, the ranking was computed according not based on the points individuals earned by themselves but rather to the points earned by their group as a whole. The four participants belonging to the highest-ranked group and the best two participants of the second highest-ranked group were assigned to hierarchy profile *A*, the other two participants of the second highest-ranked group and the participants of the third highest-ranked group to the *B* profile, and so forth for the other two hierarchy profiles. In other words, in SH treatments only individual performance mattered, whereas in CH treatments it was important to contribute to the group effort to ensure a good ranking for oneself. Before starting Phase II all participants were assigned to a hierarchy profile according to the experimental treatment condition they were assigned.

### Phase II: Cooperation in a hierarchical structure

During Phase II participants in all treatments played an Asymmetric Prisoner’s Dilemma (APD) game in which their payoffs were biased according to their hierarchy profile. Phase II was the same for all three experimental treatments and it consisted of playing five APD games of ten rounds. The exact number of rounds was unknown to participants who only knew that they had to play for at least 5 rounds. Participants were assigned to dyads to play the APD game five times. Dyads were formed using a random permutation of participants, so each individual met all hierarchy profiles once with the exception of their own hierarchy profile which they met twice. The APD game was created using the standard payoffs of a Prisoner’s Dilemma game where mutual cooperation is paid *R* = 3, mutual defection *P* = 2, cooperation to defection *S* = 1, and defection to cooperation *T* = 4. However, the actual payoffs that participants received at the end of an APD round were then multiplied by a multiplication factor *m*_*H*_ which depended on their hierarchy profile *H* = {*A,B,C,D*}, where *m*_*A*_ = 5, *m*_*B*_ = 4, *m*_*C*_ = 3, *m*_*D*_ = 2. For instance, a *B*-profile cooperator against a *C*-profile defector receives *m*_*B*_ × *S* = 4 × 1 = 4 points while the other gets *m*_*C*_ × *T* = 3 × 4 = 12 points; two cooperators of the same hierarchy profile *A* receive both *m*_*A*_ × *R* = 5 × 3 = 15 points.

After reading the detailed instructions of the experiment and answering some trial questions, participants played two repetitions of their assigned treatment, that is, (Phase I + Phase II) for SH and CH treatments and Phase II for the RH treatment. We excluded automatic answers from the analysis and analyzed only participants’ decisions in the second repetition treatment in order to consider behavior after the learning stage (during the first repetition). Before each treatment repetition, a random reshuffling of participants was performed such that participants did not play against the same participant twice. We performed SH and CH treatments during four experimental sessions each, for a total of eight sessions. The RH treatments were run during three additional sessions. All sessions included exactly 24 individuals who did not participate in any other session of the experiment, for a total of 264 subjects.

## Results

### Phase I: Hierarchy formation

**Figure 2:**
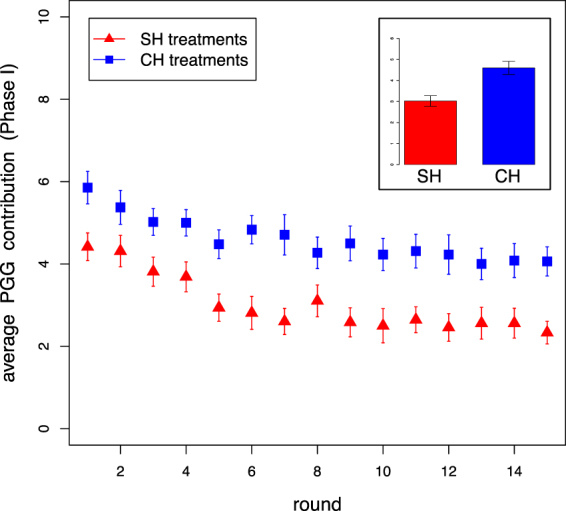
Cooperation during Phase I. Participants’ average PGG contribution as a function of the round number and over the entire phase (inset box) for both experimental treatments. Participants cooperated less in SH treatments, in which individual payoffs determine rank, compared to CH treatments, in which group earnings determine rank. (comparison of SH and CH treatment groups: MW *U* = 134, ^**^p < 0.002). Error bars represent standard error of the mean over all treatments.

We begin the presentation of our results by addressing the level of cooperation during Phase I in the SH and CH treatments; this phase was not included in the RH treatments. Figure 2 shows the average contribution over the 15 rounds of the Public Goods Game (PGG) played during Phase I. Participants cooperated ostensibly more in CH treatments than SH treatments, likely aiming to increase their group ranking position and thus obtain a higher hierarchy profile for Phase II. This is in agreement with theoretical predictions^31^. In fact, we must take into account that in SH treatments *homo economicus* individuals only benefit from maximizing their individual earnings, i.e. contributing no points to the group common pool, while in CH treatments their individual utility function corresponds to the maximization of their group payoff, i.e. contributing all the points of their round endowment. Of course, since points earned in Phase I were not converted to real money, the main assumption here is that rational individuals desire to attain the highest hierarchy profile which then allowed them to earn more points that would be converted to real money during Phase II. The fact that the observed behavior is similar to the results in paid, standard PGGs^30^ gives us confidence in our interpretation that players are seeking to maximize individual payoffs in the SH and seeking to maximize total group earnings in the CH treatment.

### Phase II: Asymmetric Prisoner’s Dilemma games

**Figure 3:**
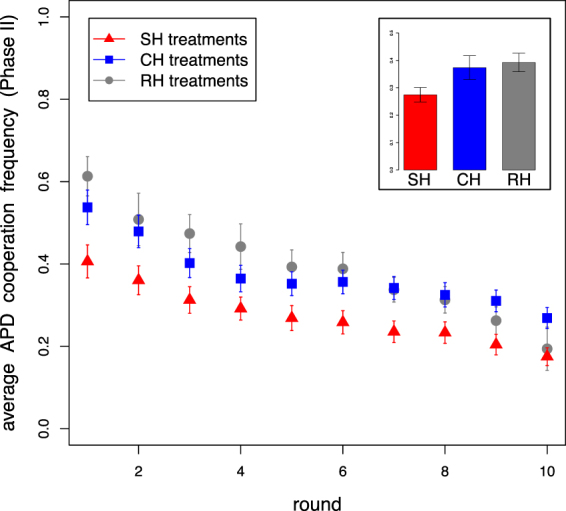
Cooperation during Phase II. Participants’ average APD cooperation frequency as a function of the round number and over the entire phase (inset box) for all experimental treatments. Participants cooperate to the same extent in CH and RH treatments and at all time less frequently in SH treatments (comparison of pairwise interaction average cooperation level distributions: SH-CH MW *U* = 34448, ^***^p < 0.001; SH-RH MW *U* = 16636, ^***^p < 0.001; CH-RH MW *U* = 20988, *p* = 0.618). Error bars represent standard error of the mean over all treatments.

We now turn to the level of cooperation during Phase II for the three experimental treatments. Fig. 3 depicts the average level of cooperation in APD games per round. We note that the number of rounds was not known by participants and the game was iterated against the same partner for ten rounds. As is generally the case in these paradigms, the cooperation level decreased for all treatments as a function of the number of rounds. Participants’ behaviors in the CH and RH treatments were similar to each other, but each different from the SH treatment (Fig. 3). This may be due to participants’ perceptions being framed differently dependent upon the way in which hierarchy profiles were obtained. We also report in Figure S5 participants’ behavior as a function of the round number separated for the five dyadic interactions (see SI Section 2).

**Figure 4:**
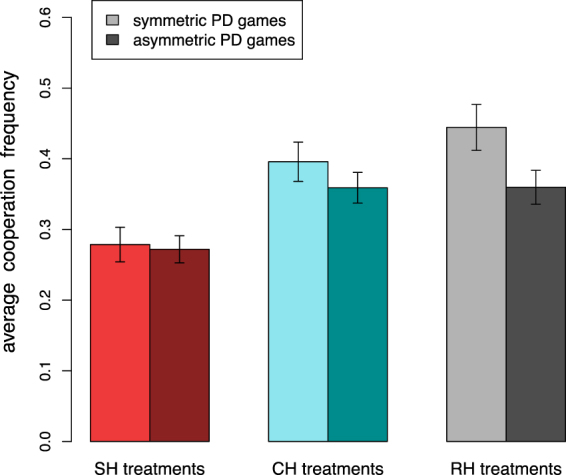
Cooperation in symmetric and asymmetric Prisoner’s Dilemma games. Participants’ average cooperation frequency for the three experimental treatments and for symmetric (lighter colors) and asymmetric (darker colors) interactions. Unbalanced interactions lead to lower levels of cooperation in RH treatments and, to a certain extent, in CH treatments (comparison of individual action distributions in symmetric and asymmetric interactions: SH treatments MW *U* = 2783500, *p* = 0.607; CH treatments MW *U* = 2866600, ^**^p < 0.010; RH treatments MW *U* = 1687000, ^***^p < 0.001). Error bars represent standard error of the mean over all dyadic interactions.

Let us now consider how cooperative behavior differs when playing against the same or a different hierarchy profile. By doing so, we can compare whether the level of cooperation in symmetric Prisoner’s Dilemma games differs from those observed in APD games or, in other words, whether the existence of a hierarchy leading to different earnings in the game for the two players has an effect. In fact, when two participants having the same hierarchy profile are paired during Phase II, the APD game can be interpreted as a symmetric one. In Fig. 4 we report the average cooperation level in the three experimental treatments for asymmetric and symmetric interactions (see also Figs S6–S7 in SI Section 2). We observe that in SH treatments participants cooperate at similar levels when playing against an individual of the same or a different hierarchy profile. On the other hand, individuals in CH and RH treatments cooperate, on average, more often in symmetric interactions with respect to asymmetric ones. However, this difference is more difficult to assess when looking at cooperation levels over the ten rounds, see Figure S6 for more details. Moreover, while cooperation levels appear similar in asymmetric pairings for CH and RH treatments, we notice that symmetric games in RH treatments show an even higher level of cooperation with respect to the one observed in CH treatments. Our finding that in two treatments asymmetry is detrimental for cooperation is in line with experiments on the asymmetric PD available in the literature^28,32^. Interestingly, the effect of the ranking in SH treatments appears to be larger than the decrease due to asymmetric interactions. In fact, cooperation levels are very similar both in symmetric and asymmetric pairings, implying that subjects were more affected by the ranking procedure than by the resulting hierarchy profiles. For further results on participants’ behavior against all hierarchy profiles during Phase II (Figure S8) and during first rounds (Figure S9) we refer the reader to Section 4 of the SI.

**Figure 5:**
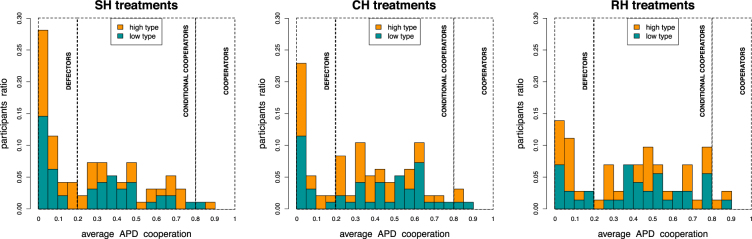
Individual cooperation during Phase II. Ratio of participants as a function of their average APD cooperation frequency and for all treatments. We define *defectors* as participants who cooperate less than 20% of the times and *cooperators* as subjects cooperating in almost all interactions (>80%). Participants having a cooperation frequency between 20% and 80% are classified as *conditional cooperators*. We observe the three participant categories in all treatments with a large prevalence of defectors and conditional cooperators with respect to pure cooperators. Hierarchy profile types are homogeneously distributed among cooperation frequencies (comparison of individual cooperation frequency distributions of low and high hierarchy profiles: SH treatments MW *U* = 1138.5, *p* = 0.923; CH treatments MW *U* = 1317, *p* = 0.224; RH treatments MW *U* = 693.5, *p* = 0.611).

A more detailed analysis provides insight on the overall individual cooperative behavior of participants during Phase II. Fig. 5 shows the proportion of participants for the three experimental treatments according to their average cooperation frequency. For the sake of simplicity, we classify hierarchy profiles into two levels, i.e. *high* and *low*, where *A* and *B* profiles are considered as high rank profiles while *C* and *D* as low rank ones, see also Fig. 1. The first conclusion one can draw from this analysis is that the behavior is rather heterogenous for all three treatments. In fact, we observe nearly the full possible range of cooperation frequencies, ranging from individuals who cooperate in almost all interactions to individuals who never do, although the latter are much more frequent in all treatments. We can thus classify players into three classes of general behavior: defectors, conditional cooperators and cooperators. We define defectors, representing the majority of participants in all treatments, as individuals who cooperate less than 20% of the time, and we define cooperators similarly as individuals who have cooperation frequency higher than 80%. We refer to the rest of the population as conditional cooperators. Furthermore, since we can find the three kinds of individuals in both hierarchy levels, it appears that the hierarchy profile of a participant does not influence the average cooperative behavior of that individual.

Finally, we analyze the individual behavior of participants when facing low or high hierarchy profiles. In order to do so, we define a measure which takes into account the influence of the hierarchy profile on the cooperation level of an individual: For each participant, we obtain her cooperation frequency *f* (between 0 and 1) against her own and the other hierarchy level, i.e. *f*_*low*_ and *f*_*high*_. For instance, a participant with *f*_*low*_ = 0.7 cooperated 70% of the time against low profile participants. We now define the *hierarchy influence H* between −1 and 1, as in Eq. 1, for each participant as the difference:

1 $$H={f}_{{\rm{high}}}-{f}_{{\rm{low}}}$$

**Figure 6:**
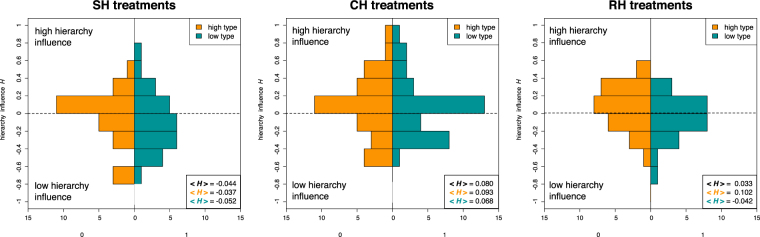
Hierarchy influence. Histograms of participants value of *H* (see text) for the three considered treatments. Results are plotted excluding pure defectors, i.e., participants with near zero H values. Mean values aggregated for all participants and for hierarchy profile classes are reported as inset. We observe more frequently low values of *H* in SH treatments (53 subjects, distribution skewness *γ*_*SH*_ = −0.097) with respect to the high values found in CH treatments (68 subjects, γ_*CH*_ = 0.327). Although negatively skewed, overall values in RH treatments (52 subjects, γ_*RH*_ = −0.506) were less scattered and more centered around zero. Again, hierarchy profile types are homogeneously distributed among all hierarchy influence values in SH and CH treatments although hierarchy influence profiles appear dependent on participants’ hierarchy type in RH treatments (comparison of hierarchy influence distributions of low and high types; SH treatments MW *U* = 381.5, *P* = 0.593; CH treatments MW *U* = 627.5, *P* = 0.547; RH treatments MW *U* = 452.5, ^*^P = 0.035).

Thus, a participant who always cooperates with low profile participants and never with high profile participants will have *f*_low_ = 1 and *f*_high_ = 0 leading to a hierarchy influence *H* = −1. Conversely, a participant who cooperates only and always with high profile participants will have *H* = 1. Finally, a participant who cooperates equally with both types has *H* = 0. Summarizing, a player who only cooperates with high profiles has a high *H* because she is affected by high profiles, and by low profiles at the same time in the opposite way, changing her behavior according to the partner’s profile. A player with hierarchy influence equal to zero cooperates to the same extent with all hierarchies. A player with negative hierarchy influence cooperates more with low profiles. Figure 6 shows the histograms of participants’ *H* for the three experimental treatments and for both hierarchy profiles. We can observe that the influence of the high hierarchy profiles is stronger in CH treatments than in the baseline scenario of RH treatments. Indeed, we find that the histogram is clearly skewed towards positive values. On the contrary, for SH treatments we observe the opposite effect, namely that the histogram is skewed towards negative values. Interestingly, we observe that low and high hierarchy profile participants appear to be equally distributed over the entire space for SH and CH treatments, again indicating that one’s own profile does not condition the subjects’ actions as much as that of the counterpart. However, we find a different hierarchy influence in RH treatments as participants tend to cooperate more frequently with partners belonging to their same hierarchy type. For further analysis on the hierarchy influence we refer the reader to Sections 5 and 6 of the SI where we present detailed scatterplots on participants’ *f*_high_ and *f*_low_ values.

## Discussion

In this paper, we have reported on experiments measuring how the way a ranking, or a hierarchy, is established in a group may affect cooperation in that group. The hierarchy formation phase (Phase I) produced results that were interesting on their own; in particular we observed that group competition increases contributions in the Public Goods Game (PGG) dilemma. While this seems to arise simply from the fact that in Collaborative Hierarchy (CH) treatments subjects must promote their group earnings, there are subtleties in the design that must be taken into account in the discussion. Indeed, in Selfish Hierarchy (SH) treatments competition among groups is always present, meaning that theoretical predictions on the classical *tragedy of the commons* problem^31^ do not completely hold. This is due to the fact that groups mostly composed of cooperators can - in terms of individual payoffs - outperform groups composed of a majority of defectors. As a result, even in SH treatments individuals belonging to generous groups may achieve higher hierarchy profiles than individuals in groups where nobody contributes. On the other hand, in CH treatments there is almost no incentive towards a non-cooperative behavior. Considering the cooperation phase when participants play the Asymmetric Prisoner’s Dilemma (APD) game, our first finding is that cooperative hierarchy formation does not lead to higher cooperation but, instead, in SH treatments subjects cooperate less. In fact, in CH treatments higher hierarchy profiles are acquired by ensuring one’s group does well, in contrast to SH treatments in which participants who defect more in their group more frequently acquire higher hierarchy profiles. The cooperative behavior in Random Hierarchy (RH) treatments represents the baseline scenario in which no framing on hierarchy profiles is introduced. It thus appears that individuals taking part in SH treatments cooperate sensibly less after performing the competitive task in their Phase I. Instead, looking at CH treatment results, performing a collaborative task during Phase I does not increase the average level of cooperation with respect to what happens in RH treatments where such a task is not present. It is interesting to note that the change in the cooperation level across treatments in Phase II is similar to what we observed in Phase I. We thus conclude that the CH treatment hierarchy assignment protocol does not increase cooperativeness but, instead, that the competition introduced in SH treatments decreases it.

What is the mechanism behind the detrimental effect of the individual hierarchy on cooperation? A first possibility is that, as in previous experiments^18^, spite leads low ranking individuals to cooperate less with high ranking ones. However, there is a crucial difference with the results reported in^18^, namely the fact that, upon successful cooperation, the higher ranked subject decides how the reward is split. This means that the reason for the low cooperativeness of lower ranked subjects could be spite, but could also be uncertainty about the benefit they would receive from their cooperation. In the setup we have studied here, there is no agency from the subjects: higher ranked participants receive more from the interaction because it is so stipulated by the game setting, and hence there is no uncertainty about the outcome of the interaction. Thus, we are left only with spite as a possible explanation of the low cooperativeness in SH treatments. However, if this factor was present, it should similarly affect the CH treatment, as there are always lower ranked and high ranked people. On the contrary, we observe cooperation with higher-ranked partners. While such behavior could be induced by the more cooperative hierarchy formation phase, we believe this is unlikely and that, as we discuss below, the mechanism at work is completely different. The key observation here is that, contrary to what was observed in^18^, a random assignment of ranking does not have any effect on cooperation as compared to CH treatments, and the observed cooperation is larger than in SH ones. We believe that this implies that subjects are not interpreting ranking as hierarchy because, as it has been already mentioned above, there is no agency from higher-ranked partners.

In the field of animal behavior, it is generally accepted that hierarchies are linked to the possibility of monopolizing access to resources and that, as a consequence, the ability of high ranking individuals to monopolize such access will predict tolerance about groupmates and the quality of social relationships in general^33,34^. We posit that the same ideas apply here, and the fact that the distribution of resources is exogenous and non-monopolizable strips ranking of its meaning. Key to this point is the realization that what a subject earns in the asymmetric PD depends, as far as hierarchy is concerned, on her own hierarchy, and not on that of her counterpart. We thus conclude that the power to affect others’ earning is crucial to establish a hierarchy, as shown by the different levels of cooperation emerging from SH and RH treatments in this experiment. The other conclusion we can draw from this observation is that high hierarchy profiles in CH treatments elicit more cooperation because of the way in which hierarchy itself is obtained, i.e., by cooperating in the PGG. High ranking is then interpreted as reputation in so far as it has been obtained by cooperating more and making the group successful. Therefore, subjects are inclined to cooperate with those identified as cooperative. Individuals have earned their rank positions through past behavior and thus their rank may be an honest signal of their future likelihood of cooperating. People are likely noting rank in their interactions and responding accordingly with their own decision to cooperate or defect in a way that maximizes their own gains. This would be compatible with the fact that, in SH treatments, participants having high hierarchy positions cheated more in the PGG, and as a consequence they were punished by experiencing less cooperation from future partners. In this treatment, participants would cooperate more with lower hierarchy profiles because they are perceived as cooperators, and therefore as ‘losers’ in the battle for hierarchy. RH treatments support this interpretation in so far as the hierarchy influence histogram is not skewed, so most people cooperate equally with partners of any level since in this treatment hierarchy does not signal previous cooperative behavior.

## Methods

All participants in the experiments reported in the manuscript signed an informed consent to participate. In agreement with the Spanish Law for Personal Data Protection, their anonymity was always preserved. This procedure was approved by the Ethics Committee of Universidad Carlos III de Madrid, the institution responsible for funding the experiment, and the experiment was subsequently carried out in accordance with the approved guidelines. The laboratory experiments were carried out with volunteers chosen among the LINEEX subject pool for the SH and CH treatments and among the IBSEN subject pool for RH treatments. The first set of experiments corresponded to eight sessions, four sessions of SH treatments and four sessions of CH treatments, and they were performed at the LINEEX experimental laboratory at different dates between the 16th and the 20th of May, 2016. The second set of experiments included three sessions of RH treatments and it was performed at the computer laboratory of the Universidad Carlos III de Madrid on November 29th, December 2nd and 14th, 2016. Participants played through a web interface specifically designed in *o*-Tree^35^ for the experiment accessible from the computers of the laboratories. At least three researchers supervised the experiment in the room, preventing any interaction among the participants. Participants were not allowed to talk or signal in any way. In order to further guarantee that potential interactions among players seated next to each other in the room did not influence results, assignment to computer stations was random and stations were isolated from each other by opaque panels. Hence there was no relationship between physical proximity and interactions in the game. Before making decisions, participants read detailed instructions and responded to a set of control questions in order to insure common understanding of the game and on the computation of payoffs. Once all questions had been answered, the first phase of the experiment began. A translation of the instructions from the original Spanish version is provided, see SI Section 1. Each session lasted about one and a half hours and included 24 participants, for a total of 264 subjects taking part in the experiment. Participants were randomly assigned to one of the three treatments. Participants received a show-up fee of 5 EUR and their personal score in Experimental Currency Units (ECUs) was converted at an exchange rate of 1 EUR = 80 ECUs at the end of the experiment. The average payoff per subject was 16.2 EUR (about 17.5 US$).

For both phases, comparisons between treatment groups were made using the nonparametric Mann-Whitney (MW) test. A nonparametric approach was appropriate for our dataset given that our data did not follow a normal distribution and we had a relatively small number of subjects per treatment. Results were considered significant when *p* < 0.05.

## Electronic supplementary material

Supplementary Information
