# Supplementary material for: Collaborative hierarchy maintains cooperation in asymmetric games
Source: Sci Rep. 2018 Mar 29;8:5375. doi: 10.1038/s41598-018-23681-z (PMC5876345; doi:10.1038/s41598-018-23681-z)
Supplement: Supplementary file 1 — Supplementary Information [file 41598_2018_23681_MOESM1_ESM.pdf]

Collaborative hierarchy maintains cooperation in asymmetric games

Supplementary Information

A. Antonioni, M. Pereda, K. A. Cronin, M. Tomassini, A. Sánchez

In Section 1 of the Supplementary Information (SI) we report translation from Spanish of the exact instructions form that participants received for the three experimental treatments, named Selfish Hierarchy (SH), Collaborative Hierarchy (CH), and Random Hierarchy (RH). SH and CH treatments involved both Phase I and Phase II. Instructions related to SH treatments only are written in red color while those for CH treatments only are written in blue color. RH treatments instructions only include those of Introduction and Phase II. A resumed version of the instructions was always available to participants during the entire experiment.

Additional results of experimental data that are not shown in the main text are presented in the following sections. We report time dependence in Section 2, participants' behavior per hierarchy profile in Section 3, and a more detailed analysis of the hierarchy influence in Section 4 (all participants) and Section 5 (excluding defectors).

1 Instructions (translated from Spanish)

Explanations for this part of the experiment

Welcome to this experiment!

You are going to take decisions that will affect your payoffs as well as the payoffs of the other participants.

Although all payoffs are expressed in Experimental Currency Units (ECUs), these points will be transformed into real money at the end of the experiment according to the following exchange rate:

80 ECUs = 1 EUR

Your total earnings in EUR will be your cumulated ECUs converted to the nearest integer-value plus a complementary show-up fee of 5 EUR.

During the experiment it is **strictly forbidden to talk to other participants**. If you have a question, please ask the assistants. If you do not comply with these rules, we will regrettably be obliged to exclude you from the experiment.

This experiment consists in two phases.

Phase I

Important note: The points you get during Phase I are not going to be converted to real money, only ECUs you get in Phase II will be converted into real money.

In this phase you will be assigned to a group of four participants, including yourself, and you will remain in the same group throughout the entire phase. This phase consists of at least ten rounds. There will be other 5 groups of 4 participants in the same situation of your group.

At the beginning of each round, you will be provided with an endowment of ten points. Then, you have to make a decision on how many points (0, 2, 4, 6, 8, 10) of this endowment you want to invest in a common pool. The points invested by all the participants in your group will be added together and multiplied by two. Finally, this common pool will be shared equally among all the participants in your group. You have 30 seconds to take this decision, otherwise the software will take a random decision at your place.

At the end of this phase you will be assigned to a profile according to the number of points you [and your group] earned. These profiles (A, B, C, D) will be assigned as follows.

SH treatment instructions (Phase I)

As Fig. S1 shows, at the end of Phase I all the 24 participants in the experiment will be ordered according to the points they have gained during that phase, where the highest-earning participant is represented by a **1** and the lowest-earning participant by a **24**.

Profile types for Phase II are assigned as follows: the six highest-earning participants to Profile **A** (1–6), the next six participants to Profile **B** (7–12), the next six participants to Profile **C** (13–18), and the last six participants to Profile **D** (19–24), as shown in S1.

1	2	3	4	5	6	Tipo A
7	8	9	10	11	12	Tipo B
13	14	15	16	17	18	Tipo C
19	20	21	22	23	24	Tipo D

Figure S1: Selfish Hierarchy structure.

For your convenience, a resumed version of these instructions will be available to you during the entire experiment.

Example:

In the below figure the 24 participants have been ordered according to their results obtained during Phase I.

The participant with number 17 is the one with the highest earnings, i.e. 43 points (left-most figure) and obtains the profile type **A** for Phase II (right-most figure). The participant with number 4 is the one with the lowest earnings, i.e. 15 points and obtains the profile type **D** for Phase II.

Participante 17 43 puntos	Participante 11 42 puntos	Participante 1 40 puntos	Participante 12 38 puntos	Participante 22 37 puntos	Participante 8 37 puntos	Tipo A	Participante 17 Tipo A	Participante 11 Tipo A	Participante 1 Tipo A	Participante 12 Tipo A	Participante 22 Tipo A	Participante 8 Tipo A
Participante 6 36 puntos	Participante 23 36 puntos	Participante 15 35 puntos	Participante 10 34 puntos	Participante 2 30 puntos	Participante 24 30 puntos	Tipo B	Participante 6 Tipo B	Participante 23 Tipo B	Participante 15 Tipo B	Participante 10 Tipo B	Participante 2 Tipo B	Participante 24 Tipo B
Participante 3 27 puntos	Participante 19 26 puntos	Participante 5 25 puntos	Participante 18 25 puntos	Participante 13 25 puntos	Participante 20 24 puntos	Tipo C	Participante 3 Tipo C	Participante 19 Tipo C	Participante 5 Tipo C	Participante 18 Tipo C	Participante 13 Tipo C	Participante 20 Tipo C
Participante 14 23 puntos	Participante 9 22 puntos	Participante 7 22 puntos	Participante 21 18 puntos	Participante 16 17 puntos	Participante 4 15 puntos	Tipo D	Participante 14 Tipo D	Participante 9 Tipo D	Participante 7 Tipo D	Participante 21 Tipo D	Participante 16 Tipo D	Participante 4 Tipo D

Figure S2: Selfish Hierarchy example.

CH treatment instructions (Phase I)

As Fig. S3 shows, at the end of Phase I all the 24 participants in the experiment will be ordered according to the points their group has gained and also according to their individual earnings, where the first group (white group in Fig. S3) is the group that gained the most, and the last group (the darker one in Fig. S3) is the group that gained the least.

Profile types for Phase II are assigned as follows: the highest-earning group and the two highest-earning people of the second highest-earning group to Profile **A**, the two lowest-earning people of the second highest-earning group and the third group to Profile **B**, the forth highest-earning group and the two highest-earning people of the fifth highest-earning group to Profile **C**, and the two lowest-earning people of the fifth highest-earning group and the lowest-earning group to Profile **D**.

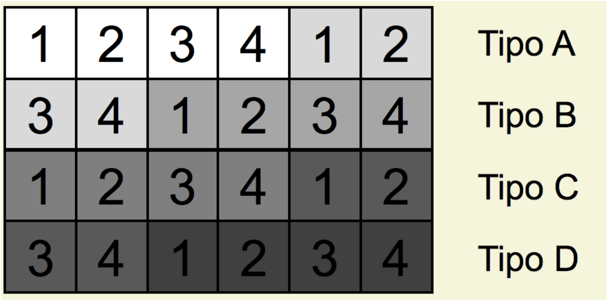

Figure S3: Collaborative Hierarchy structure.

For your convenience, a resumed version of these instructions will be available to you during the entire experiment.

Example:

In the below figure the 24 participants have been ordered according to their results obtained during Phase I. The white-colored group is the one with the highest earnings, i.e. 163 points. Within the white-colored group, the participant with number 17 is the one with the highest individual earnings, i.e. 43 points. All the participants belonging to the white-colored group obtain the profile type **A** (see right-most figure) for Phase II.

The second group is formed by participants with numbers 6, 8, 22 and 23. The participant with the number 22 is the one with highest individual earnings with the belonging group while participant with the number 23 is the one who individually gained less. Consequently, participants with numbers 8 and 22 obtain the profile type **A**, and participants with numbers 6 and 23 obtain the profile type **B**.

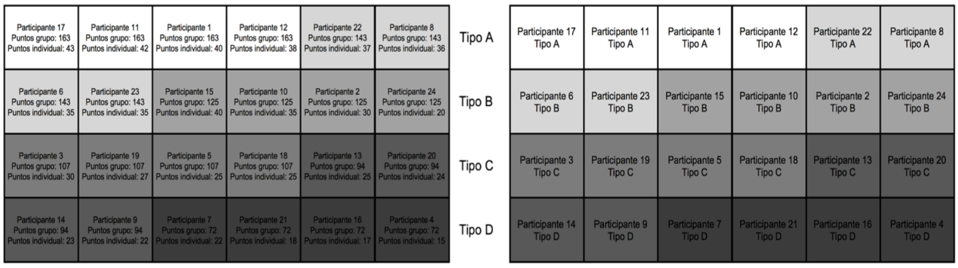

Figure S4: Collaborative Hierarchy example.

Phase II

This phase consists of five stages. Each stage consists of at least five rounds.

In each stage, you will be paired to a randomly selected participant, henceforward, your *partner*. You and your partner have to choose an *action* among the two following options:

X or **Y**

These decisions will affect your and your partner's earnings.

You and your partner will be assigned to a multiplication factor m according to the profile type you obtained in Phase I. We call here your multiplication factor as m_1 and your partner's multiplication factor as m_2. In the following table, you can see which multiplication factor corresponds to each profile.

Profile type	A	B	C	D
Multiplication factor m	5	4	3	2

Now we explain the payoffs for each possible combination of your action and your partner's action, considering your multiplication factor as m_1 and your partner's multiplication factor as m_2:

- You choose **X**, your partner chooses **X**:
  you gain **3**$\times m_1$ **ECUs** and your partner gets **3**$\times m_2$ **ECUs**.
- You choose **X**, your partner chooses **Y**:
  you gain **1**$\times m_1$ **ECUs** and your partner gets **4**$\times m_2$ **ECUs**.
- You choose **Y**, your partner chooses **X**:
  you gain **4**$\times m_1$ **ECUs** and your partner gets **1**$\times m_2$ **ECUs**.
- You choose **Y**, your partner chooses **Y**:
  you gain **2**$\times m_1$ **ECUs** and your partner gets **2**$\times m_2$ **ECUs**.

For your convenience, a resumed version of these instructions will be available to you during the entire experiment.

Examples (Phase II):

Your profile type is **A** and your partner's one is **C**. You both choose **X**. You gain $3 \times 5 = 15$ ECUs while your partner gains $3 \times 3 = 9$ ECUs.

Your profile type is **B** and your partner's one is **A**. You choose **X** and your partner chooses **Y**. You gain $1 \times 4 = 4$ ECUs while your partner gains $4 \times 5 = 20$ ECUs.

Trial questions

Before starting the actual experiment we would like to be sure that you and everybody else has correctly understood the instructions. To this end, please answer the questions that will appear on your screen. When you are done with a question click the "OK" button at the bottom of the screen.

(*Participants answer the following trial questions after reading the instructions. Wrong answers were corrected by the software showing the participants the correct answer and related explanations. Participants were able to begin the experiment only answering to all questions.*)

1. (*Phase I only*) How many points do you receive as endowment at the beginning of a round during Phase I?
   Correct answer: **10** points.

2. (*Phase I only*) Suppose that you contribute zero points to the common pool and that the sum of all contributions in the common pool from all participants in your group is 20.
   Which is your gain at the end of this round?
   Correct answer: **20** points.

3. (*Phase I only*) Suppose that you contribute 10 points to the common pool and that the sum of all contributions in the common pool from all participants in your group is 30.
   Which is your gain at the end of this round?
   Correct answer: **15** points.

4. (*Phase I only*) Suppose that at the end of Phase I you are the **12th** highest-earning participant among the 24 participants.
   Which will your profile type be for Phase II?
   Correct answer: **B**.

5. (*Phase I only*) Suppose that at the end of Phase I you are the **19th** highest-earning participant among the 24 participants.
   Which will your profile type be for Phase II?
   Correct answer: **D**.

6. (*Phase I only*) Suppose that at the end of Phase I your group is the **third** highest-earning one and that you are the **third** highest-earning participant within your group.
   Which will your profile type be for Phase II?
   Correct answer: **B**.

7. (*Phase I only*) Suppose that at the end of Phase I your group is the **fifth** highest-earning one and that you are the **second** highest-earning participant within your group.
   Which will your profile type be for Phase II?
   Correct answer: **C**.

8. (*related to Phase II*) Suppose that your profile type is **B** and your partner's one is **C**. You choose **X** and your partner chooses **Y**.
   Which is your gain at the end of this round?
   Correct answer: **4** points.

9. (*related to Phase II*) Suppose that your profile type is **D** and your partner's one is **A**. You choose **Y** and your partner chooses **X**.
   Which is your gain at the end of this round?
   Correct answer: **8** points.

10. (*related to Phase II*) Suppose that your profile type is **C** and your partner's one is **D**. You choose **Y** and your partner chooses **Y**.
    Which is your gain at the end of this round?
    Correct answer: **6** points.

11. (*related to Phase II*) Suppose that your profile type is **A** and your partner's one is **B**. You choose **X** and your partner chooses **X**.
    Which is your gain at the end of this round?
    Correct answer: **15** points.

2 Time dependence

In Fig. S5 we plot the average cooperation level during Phase II for the three treatments as a function of the round number. Participants interact with the same partner for ten rounds before changing for a new one. We observe that in all treatments and for all of the five dyadic interactions the global pattern is a decrease in the cooperation level, with a high pick in the first few rounds with a new partner. Cooperation levels are almost always higher in RH and CH treatments with respect to SH treatments, see Fig. 2 of the main text for more details on statistics. It appears that average cooperation levels during the five pairwise interactions tend to slightly increase in all treatments.

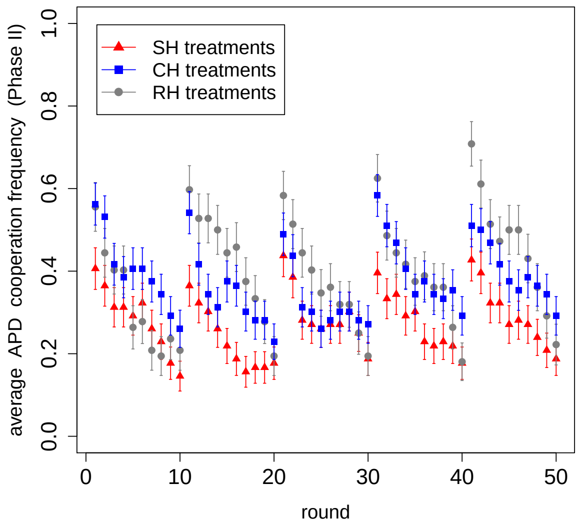

Figure S5: **Cooperation per round number**. Average APD cooperation frequency for the three treatments as a function of the round number. Error bars represent standard error of the mean.

In Fig. S6 we plot the average cooperation level during Phase II for symmetric and asymmetric games and for the three treatments. Asymmetric interactions are considered the ones between participants having a different hierarchy profile type. We observe that in all treatments the global pattern is a decrease in the cooperation level. However, while cooperation levels for symmetric and asymmetric interactions in SH and CH treatments are rather undistinguishable, participants in RH treatments cooperate sensibly less with different-profile participants with respect to partners having the same hierarchy profile type.

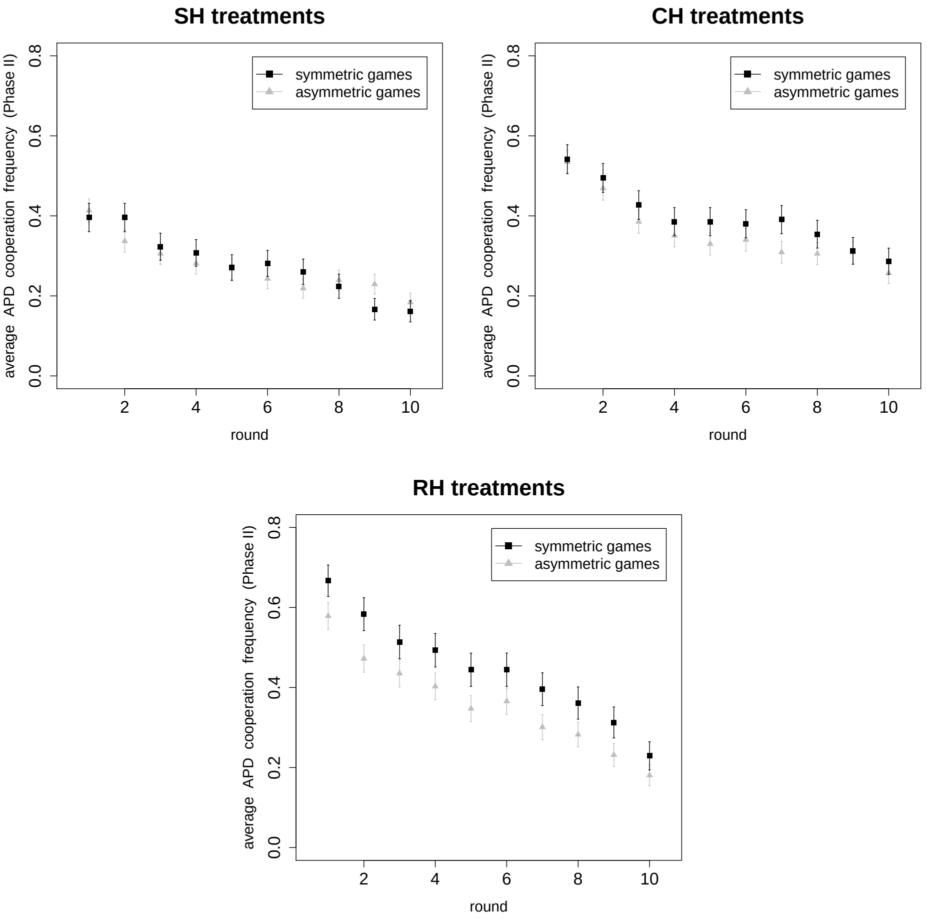

Figure S6: **Cooperation in PD games**. Average cooperation frequency in asymmetric and symmetric games for the three treatments as a function of the round number. Error bars represent standard error of the mean.

In Fig. S7 we plot the average cooperation level during Phase II for the four hierarchy profile types and for the three treatments as a function of the round number. Again, we observe that in all treatments the global pattern is a decrease in the cooperation level. However, we cannot assess any clear pattern among different hierarchy profile types although cooperation levels in RH treatments look more scattered than those in SH and CH treatments. In particular, in RH treatments, A-profile participants start cooperating more often than others during initial rounds while D-profile participants defect more frequently during last rounds.

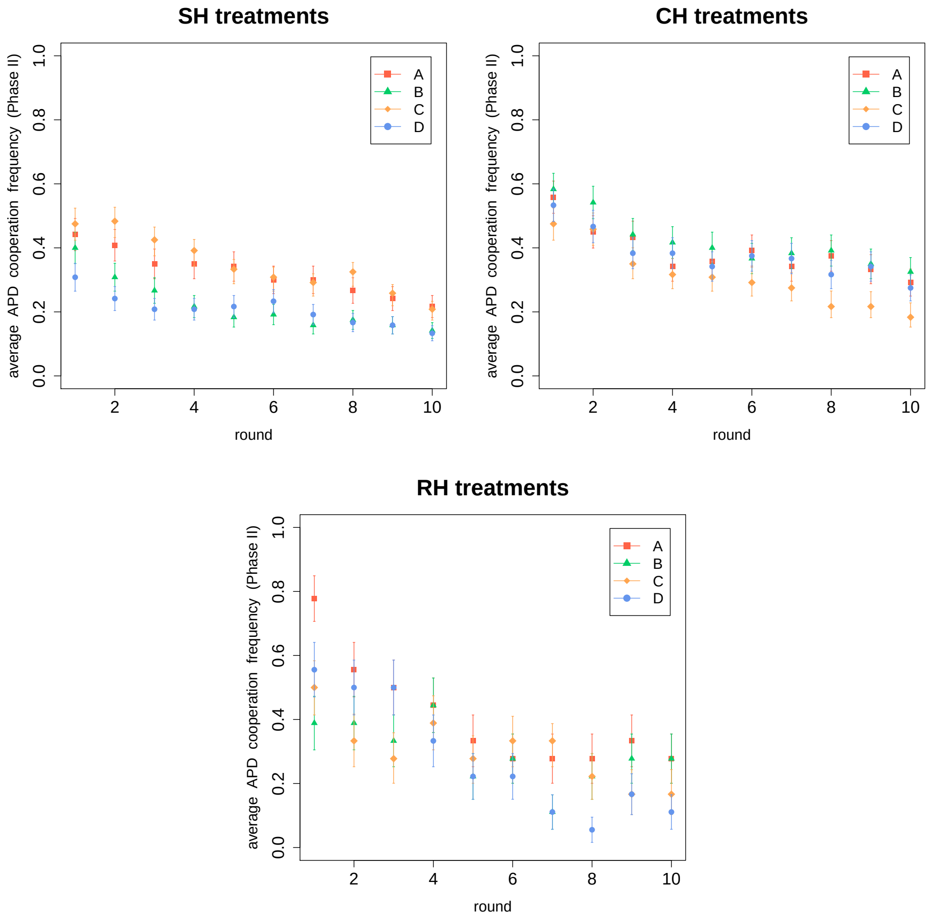

Figure S7: **Cooperation per hierarchy profile**. Average cooperation frequency for the four hierarchy types and for the three treatments as a function of the round. Error bars represent standard error of the mean.

3 Hierarchy profile behavior

In Fig. S8 we plot the average cooperation level during Phase II for the four hierarchy profile types and for the three treatments. As in Fig. S7, we cannot assess any clear pattern among different hierarchy profiles when looking at the four types.

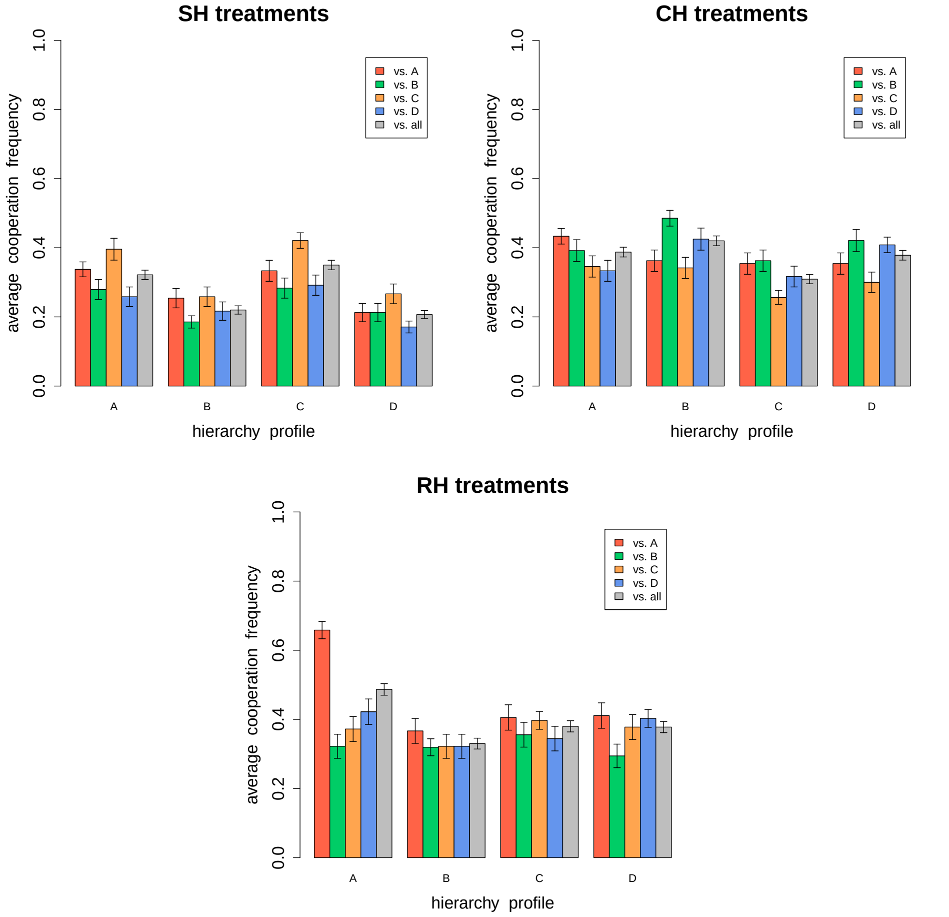

Figure S8: **Global cooperation per hierarchy profile**. Average cooperation frequency for the four hierarchy types and for the three treatments as a function of partner's hierarchy profile. Colored bars represent the four hierarchy types of the partner while gray bars are the global cooperation frequency per hierarchy profile, i.e. cumulated against all profiles. Error bars represent standard error of the mean.

In Fig. S9 we plot the average cooperation level during first rounds in Phase II for the four hierarchy profile types and for the three treatments. Similarly to Figs. S7- S8, we cannot assess any clear pattern among different hierarchy profile types although A-profile participants in RH treatments cooperate sensibly more frequently than others.

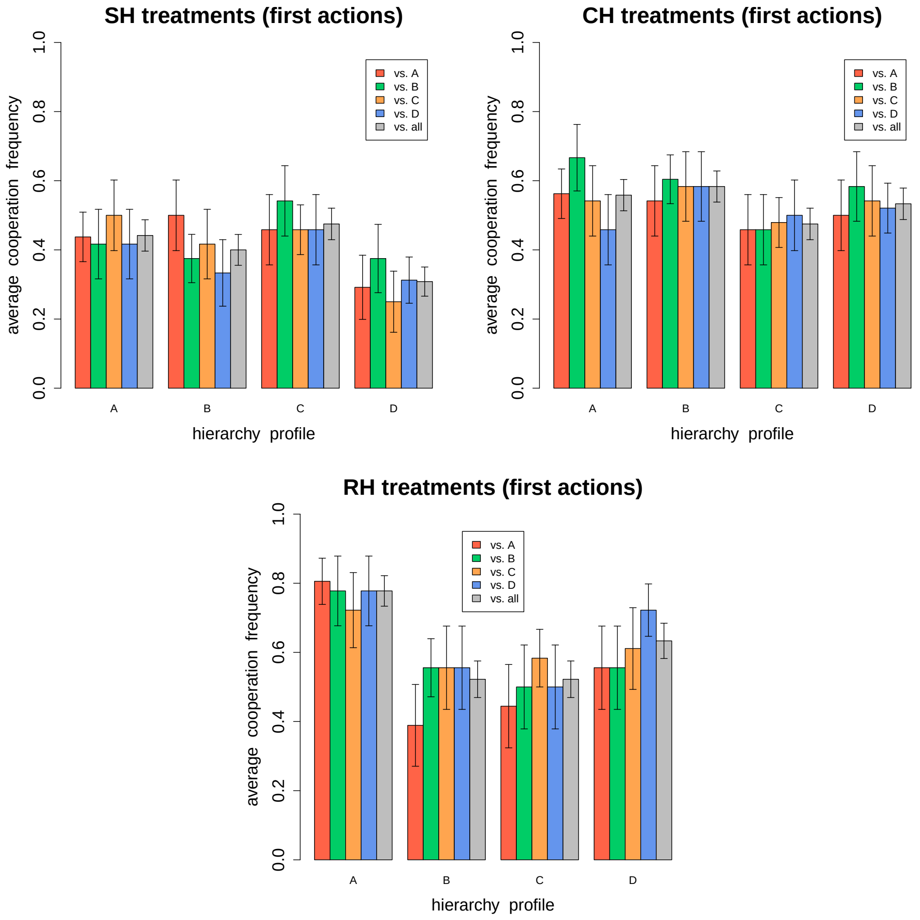

Figure S9: **Cooperation during first interactions**. Average cooperation frequency for the four hierarchy types and for the three treatments as a function of partner's hierarchy profile during first rounds. Colored bars represent the four hierarchy types of the partner while gray bars are the global cooperation frequency per hierarchy profile, i.e. cumulated against all profiles. Error bars represent standard error of the mean.

4 Hierarchy influence analysis

In Figs. S10, S11 and S12 we present a scatterplot for the three treatments to show the hierarchy influence $H = f_{high} - f_{low}$ value for each participant, where f_{low} (f_{high}) is the cooperation frequency against a low (high) profile participant. Each point represents a participant and the value H plotted in Fig. 6 of the main text can be seen as the distance from the point to the bisector line. When $H > 0$ participants stand in the upper-left section.

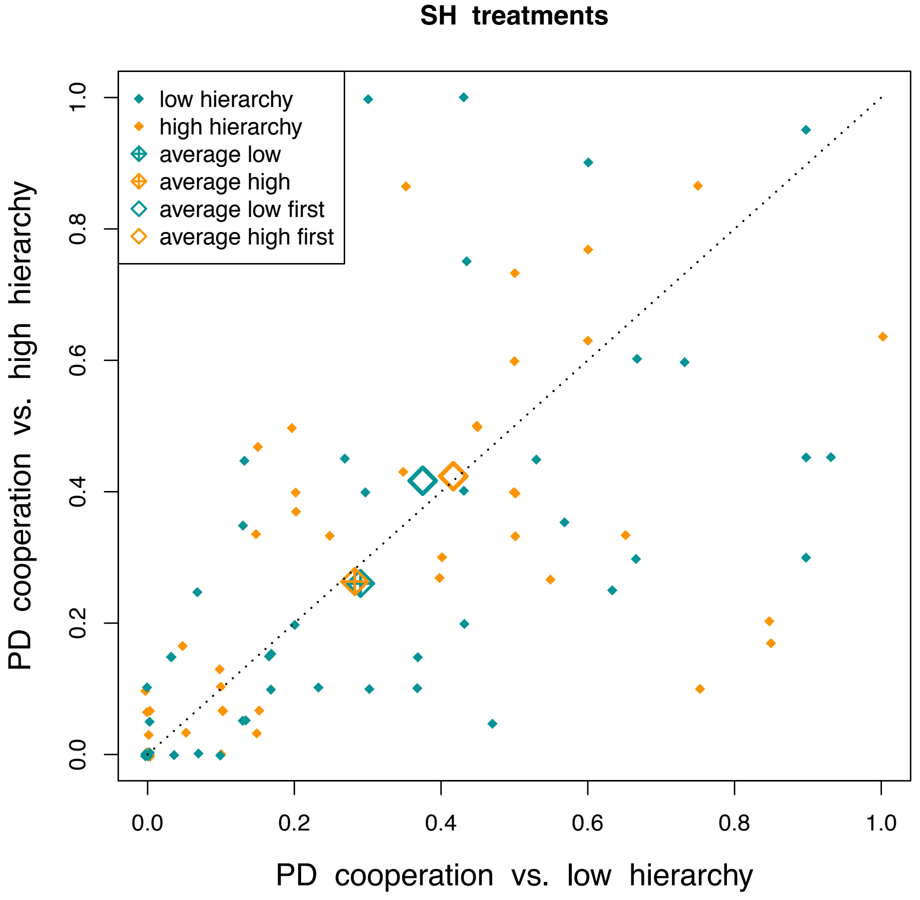

Figure S10: **Hierarchy influence per hierarchy type**. Each point represents a participant during Phase II in SH treatments where its coordinates are participant's cooperation frequency f_{high} (y-axis) against high hierarchy profiles, i.e. A- and B-type, and participant's cooperation frequency f_{low} (x-axis) against low hierarchy profiles, i.e. C- and D-type. We present mean values per hierarchy profile for all participants cumulated over all rounds (crossed diamonds) and during first actions (empty diamonds).

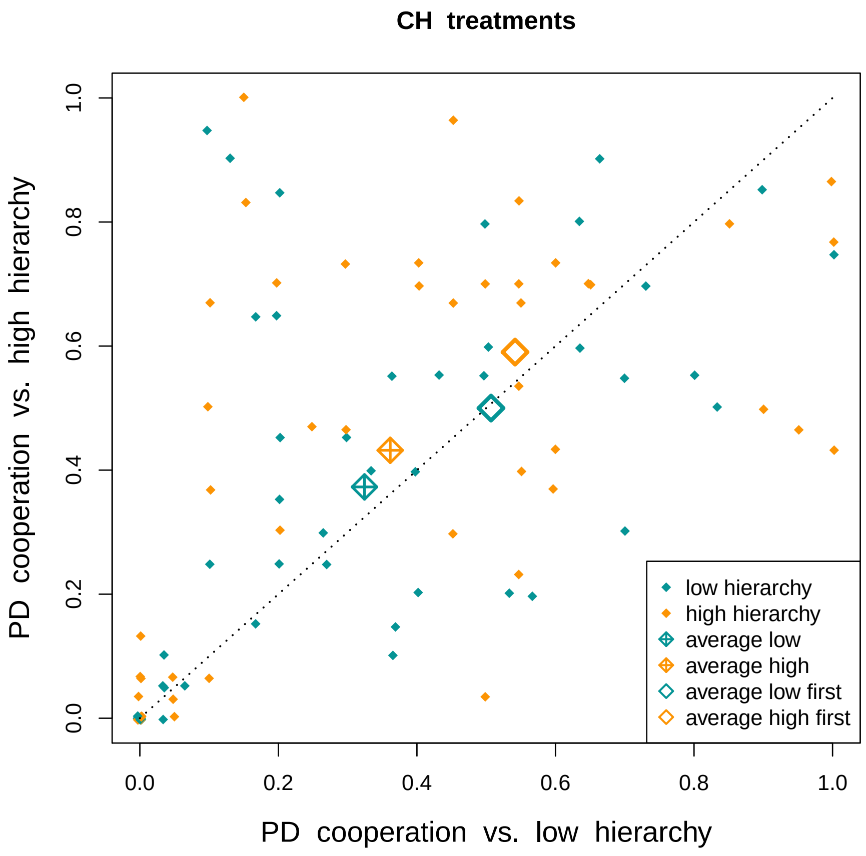

Figure S11: **Hierarchy influence per hierarchy type**. Each point represents a participant during Phase II in CH treatments where its coordinates are participant's cooperation frequency f_{high} (y-axis) against high hierarchy profiles, i.e. A- and B-type, and participant's cooperation frequency f_{low} (x-axis) against low hierarchy profiles, i.e. C- and D-type. We present mean values per hierarchy profile for all participants cumulated over all rounds (crossed diamonds) and during first actions (empty diamonds).

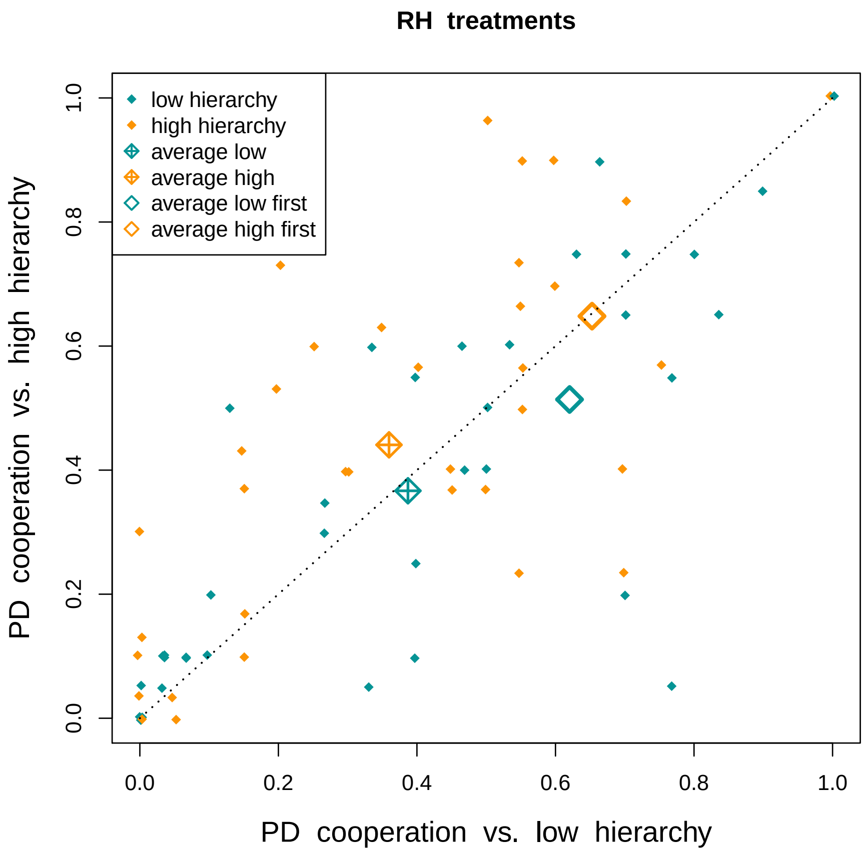

Figure S12: **Hierarchy influence per hierarchy type**. Each point represents a participant during Phase II in RH treatments where its coordinates are participant's cooperation frequency f_{high} (y-axis) against high hierarchy profiles, i.e. A- and B-type, and participant's cooperation frequency f_{low} (x-axis) against low hierarchy profiles, i.e. C- and D-type. We present mean values per hierarchy profile for all participants cumulated over all rounds (crossed diamonds) and during first actions (empty diamonds).

5 Hierarchy influence analysis (excluding defectors)

In Figs. S13, S14 and S15 we present the scatterplots of Figs. S10, S11 and S12 excluding *defector* participants from the analysis. All mean values are thus computed in a different way while the other points remain the ones from previous section. Here, we can observe more clearly that average values for high and low profile participants are rather different (more discussion on the hierarchy influence in the main text). These results are related to those of Fig. 6 of the main text.

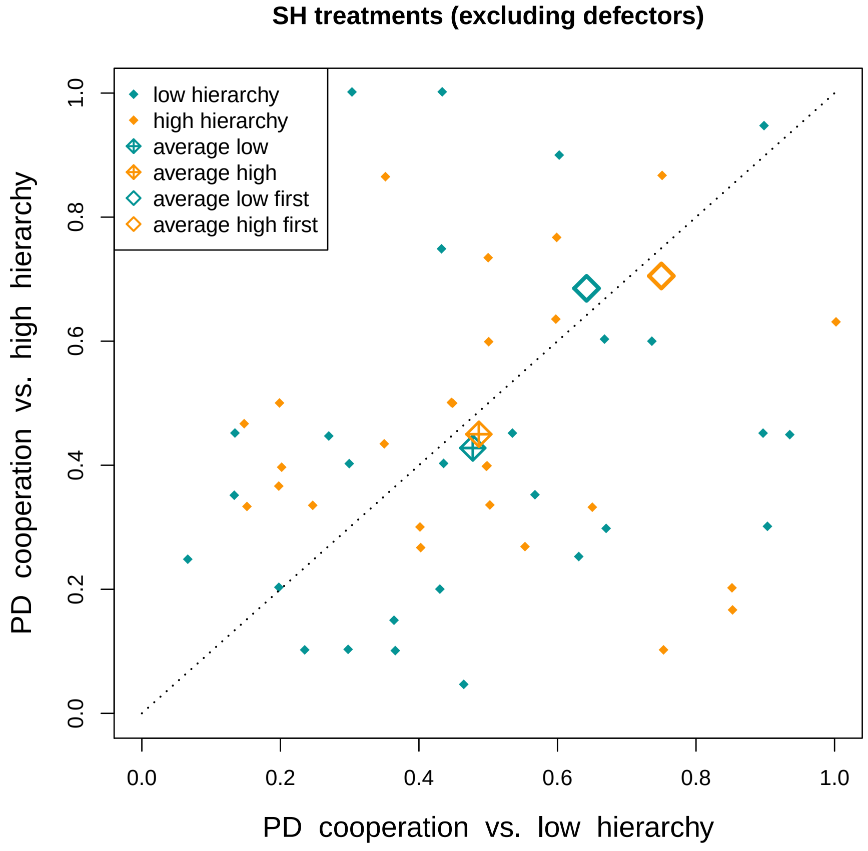

Figure S13: **Hierarchy influence per hierarchy type**. Each point represents a participant during Phase II in SH treatments where its coordinates are f_{high} (y-axis) and f_{low} (x-axis). We plot mean values per hierarchy profile for all participants cumulated over all rounds (crossed diamonds) and during first actions (empty diamonds). We exclude defector participants, i.e. subjects who cooperated, on average, less than 20% of the time.

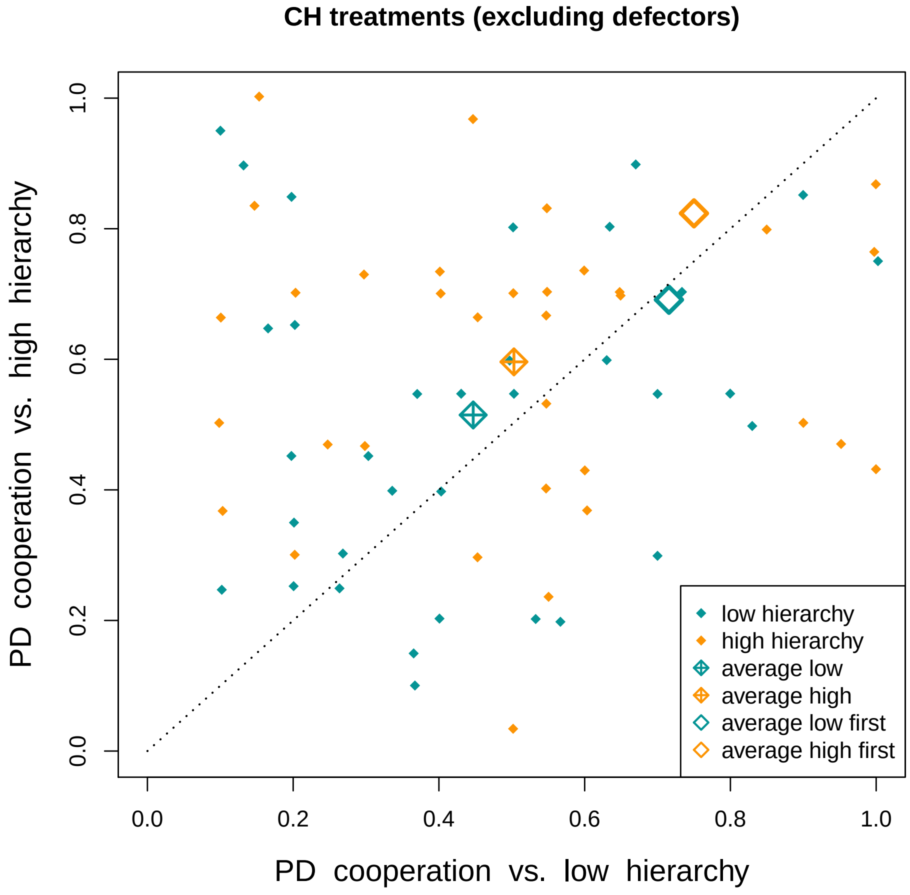

Figure S14: **Hierarchy influence per hierarchy type**. Each point represents a participant during Phase II in CH treatments where its coordinates are f_{high} (y-axis) and f_{low} (x-axis). We plot mean values per hierarchy profile for all participants cumulated over all rounds (crossed diamonds) and during first actions (empty diamonds). We exclude defector participants, i.e. subjects who cooperated, on average, less than 20% of the time.

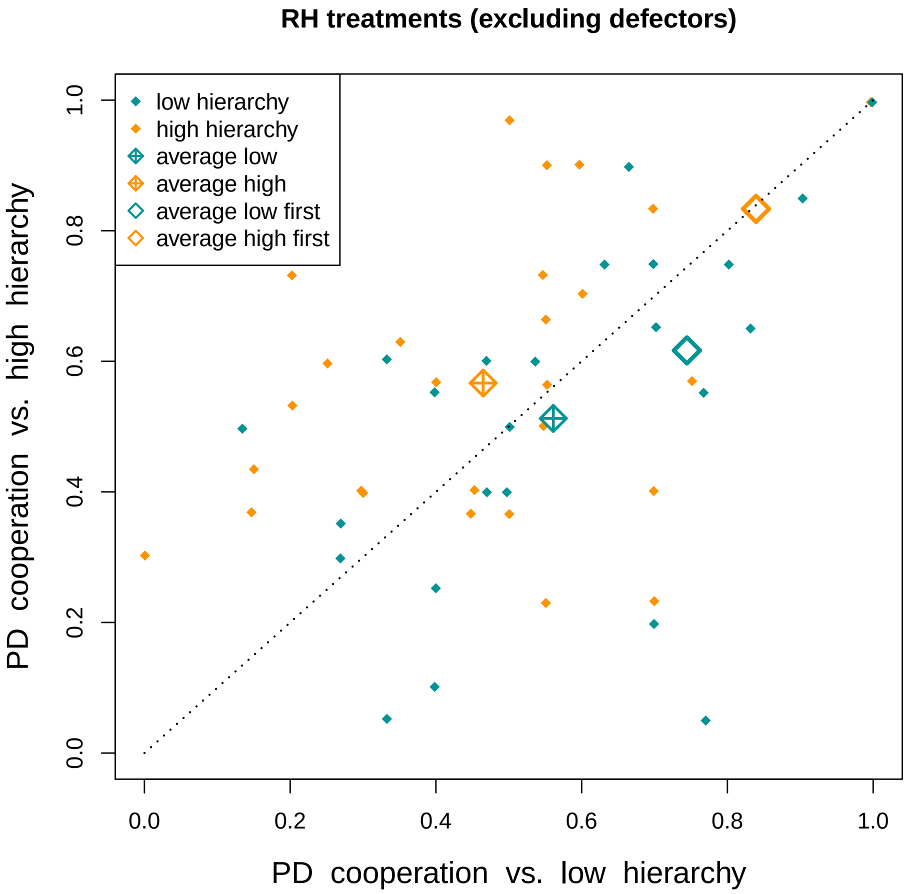

Figure S15: **Hierarchy influence per hierarchy type**. Each point represents a participant during Phase II in RH treatments where its coordinates are f_{high} (y-axis) and f_{low} (x-axis). We plot mean values per hierarchy profile for all participants cumulated over all rounds (crossed diamonds) and during first actions (empty diamonds). We exclude defector participants, i.e. subjects who cooperated, on average, less than 20% of the time.
